# Redox‐Dependent Activation of Matrix Metalloproteinases Regulates Influenza A Virus Replication in Neuronal Cells

**DOI:** 10.1155/ijm/7586794

**Published:** 2026-05-18

**Authors:** Carla Prezioso, Marta De Angelis, Dolores Limongi, Anna Maria Marinelli, Flavio Frezza, Lucia Nencioni, Anna Teresa Palamara, Paola Checconi

**Affiliations:** ^1^ Department for the Promotion of Human Sciences and Quality of Life, San Raffaele University, Rome, Italy, unisr.it; ^2^ Laboratory of Microbiology, IRCCS San Raffaele Roma, Rome, Italy; ^3^ Department of Public Health and Infectious Diseases, Laboratory Affiliated to Istituto Pasteur Italia-Fondazione Cenci Bolognetti, Sapienza University of Rome, Rome, Italy, uniroma1.it; ^4^ Laboratory of Virology, Department of Molecular Medicine, Sapienza University, Rome, Italy, uniroma1.it; ^5^ Department of Infectious Diseases, Istituto Superiore di Sanità, Rome, Italy, iss.it

**Keywords:** influenza virus, matrix metalloproteinases, neuronal cells, oxidative stress, redox modulators

## Abstract

**Background:**

Influenza A virus (IAV) is a major respiratory pathogen with the potential to invade the central nervous system (CNS), leading to neurological complications. However, the mechanisms underlying IAV neurotropism and its impact on neuronal cells remain poorly understood. This study is aimed at establishing a reliable in vitro model using differentiated SH‐SY5Y human neuroblastoma cells to investigate IAV infection in the CNS, with a specific focus on oxidative stress and matrix metalloproteinases (MMPs) production.

**Methods:**

Differentiated SH‐SY5Y cells were infected with Influenza A/NWS/33 (H1N1) strain. Redox state was assessed by measuring intracellular glutathione (GSH) levels, Glutaredoxin 1 (Grx1) expression, and Prdx1 release. The activation of oxidative stress‐responsive MMPs (MMP‐2, MMP‐9) was analyzed via western blot, gelatin zymography, and qRT‐PCR. The effect of MMPs inhibition on viral replication was evaluated using Batimastat (BB‐94) and siRNA‐mediated downmodulation.

**Results:**

IAV efficiently infects and replicates in differentiated SH‐SY5Y cells, inducing oxidative stress, as evidenced by decreased GSH levels, increased glutathionylated proteins and Grx1 expression, and Prdx1 release. Additionally, IAV infection enhances the production and activation of MMP‐2 and MMP‐9, a process regulated by redox balance, as demonstrated by the inhibitory effects of redox modulators. Finally, postinfection treatment with BB‐94, an MMPs inhibitor, and siRNA‐mediated downmodulation significantly reduces viral replication and infectivity.

**Conclusions:**

These findings demonstrate that IAV infection modulates neuronal redox balance and promotes MMPs activation, fostering a cellular environment that enhances viral replication and spread within neuronal‐like cells.

## 1. Introduction

Influenza viruses are major respiratory pathogens that cause significant morbidity and mortality worldwide [1]. In fact, even if these viral infections are often self‐resolving and limited to the upper airways, they can lead to severe respiratory illness, such as pneumonia, in vulnerable populations, particularly in elderly and immunocompromised individuals [2]. Moreover, extrapulmonary complications, involving many other organs and systems, including cardiovascular and nervous ones, have been reported [3]. Although it is well known that influenza virus has a marked tropism for respiratory tract, it can reach the central nervous system (CNS) and cause encephalopathy, or other damages, often under‐recognized and whose mechanisms are still unclear [4].

Critical factors contributing to the severity of influenza infection are the host′s cell responses, as redox‐sensitive pathways induction [5], with the activation and/or upregulation of enzymes, including kinases, redoxins, and matrix metalloproteinases (MMPs) [6–8]. The interplay between the influenza virus, particularly Influenza A virus (IAV), and redox state has been extensively characterized in epithelial cells. IAV infection is known to disrupt the intracellular redox balance between reactive oxygen species (ROS) and antioxidant defenses, leading to oxidative stress. Induction of ROS production can activate various signaling pathways, such as the caspase cascade that results in apoptotic cell death, damaging lung tissue [9]. ROS can trigger an inflammatory cascade too, with the production of proinflammatory cytokines and chemokines; this cytokine storm exacerbates lung injury, contributing to the severity of the disease [10].

Among redox‐sensitive enzymes, MMPs are a group of zinc‐dependent endopeptidases involved in the degradation of extracellular matrix components. They play essential roles in physiological processes such as tissue remodeling, wound healing, and embryogenesis [11]. However, MMPs are also implicated in various pathological conditions, including cancer, cardiovascular diseases, arthritis, and multiple sclerosis, as shown by increased MMP‐9 levels in patients under natalizumab treatment [11, 12]. MMP‐9, along with MMP‐2, are of particular interest due to their involvement in inflammatory responses and tissue destruction during infections [13, 14]. MMP‐9, in particular, has been shown to degrade Type IV collagen, a major component of the basement membrane, thereby affecting the integrity of tissues and facilitating the spread of pathogens [15, 16]. It has been demonstrated also that virus‐induced degradation of the basement membrane by MMP‐9 can compromise the blood brain barrier (BBB), potentially leading to neurological consequences [16, 17].

Studies have demonstrated that respiratory virus infections can lead to the significant upregulation of MMP‐9, whose plasma levels, for instance, were found increased in COVID‐19 patients [18]. Research on Sendai virus, revealed marked increases in MMP‐9 expression in virus‐infected rat lungs [19], as well as in IAV‐infected mice lungs [20]. Moreover, in fetal membrane cells, IAV infection has been shown to enhance MMP‐9 expression and activate MMP‐2, which may contribute to adverse pregnancy outcomes [21]. This highlights the broader implications of MMPs regulation beyond the respiratory system, indicating a systemic impact of influenza infections.

The interplay between influenza virus, oxidative stress, and MMPs underscores the complexity of the host response to viral infections. Although oxidative stress is a crucial mediator of influenza‐induced lung injury, it also regulates the expression of MMPs, which can further exacerbate tissue damage. On the other hand, given the potential for influenza to invade the CNS and the role of MMPs in disrupting the BBB, it is crucial to study these interactions in a neuronal model.

Therefore, the aim of this study was to establish a reproducible in vitro model using differentiated SH‐SY5Y human neuroblastoma cells to investigate IAV infection within the CNS, with a specific focus on oxidative stress and the production of MMPs.

## 2. Materials and Methods

### 2.1. SH‐SY5Y Cell Culture and Differentiation

SH‐SY5Y human neuroblastoma cells were cultured in DMEM with 10% FBS, 0.3 mg/mL glutamine, 100 U/mL penicillin, and 100 *μ*g/mL streptomycin at 37°C and 5% CO_2_, subcultured at 70%–80% confluency.

Neuronal differentiation was induced using DMEM with 1× nonessential amino acid (NEAA), 1× sodium pyruvate, 10% FBS, then lowered to 1%, plus 10 *μ*M retinoic acid (RA). Medium was refreshed every 3 days and differentiation was monitored microscopically and further confirmed at the molecular level by assessing *β*III‐tubulin (TUBB3) mRNA expression via quantitative real‐time PCR (qRT‐PCR) and by analyzing *β*III‐tubulin protein expression through western blot. Experiments began after 6 days of differentiation, according to published protocol [22].

### 2.2. Virus Infection and Titration

Influenza A/NWS/33 H1N1 (NWS) was propagated in 10‐day embryonated eggs and harvested after 48 h. SH‐SY5Y cells were infected at 0.1 m.o.i. for 1 h, washed, and incubated with 2% FBS medium for 24 h. Viral RNA in supernatants was quantified by qRT‐PCR and the infectivity by measuring the Tissue Culture Infectious Dose 50% (TCID50) [23].

### 2.3. Reagents Treatment

R,R ^′^‐2‐Acetylamino‐3‐[4‐(2‐acetylamino‐2carboxyethylsulfanylthiocarbonylamino) phenylthiocarbamoylsulfanyl] propionic acid hydrate (2‐AAPA, Sigma‐Aldrich), was added to SH‐SY5Y cells 20 min before infection, 100 *μ*M [24]. GSH‐C4 (Redox‐Co) was added postinfection at 5 and 10 mM [25] Batimastat (BB‐94, SelleckChemicals) was administered 1 h before infection, during infection, that is, with the virus for the 1 h viral adsorption time, and after infection, that is, after the 1 h viral adsorption time and kept for the next 24 h, at 25 *μ*M. 2‐AAPA and BB‐94 were dissolved in DMSO and then diluted to the final concentration in the cell culture medium; DMSO concentration did not exceed 0.2%.

### 2.4. MTT Assay

Cell viability under reagents treatment was assessed by the 3‐(4,5‐dimethyl‐2‐thiazolyl)‐2,5 diphenyl tetrazolium bromide (MTT) assay. Differentiated SH‐SY5Y cells were treated with BB‐94 at concentrations ranging from 1 to 100 *μ*M under the same experimental conditions used for infection experiments. After 24‐h treatment, MTT solution was added and incubated for 4 h at 37°C. Formazan crystals were dissolved in DMSO, and absorbance was measured at 570 nm. Cell viability was expressed as a percentage relative to vehicle‐treated control cells.

### 2.5. Glutathione Assay

Intracellular GSH was measured with the glutathione assay kit (ADI‐900‐160 Enzo) after deproteinization with metaphosphoric acid of cell lysates. Protein content was measured with the DC protein assay (Bio‐Rad) and GSH level expressed as nmol/mg protein.

### 2.6. Western Blot Analysis

Cells were lysed in lysis buffer with PMSF and protease inhibitor mixture (Sigma‐Aldrich). Protein was quantified with DC protein assay (Bio‐Rad) and analyzed by SDS‐PAGE followed by western blot. Proteins were visualized using the following primary and HRP‐conjugated secondary antibodies: anti‐*β*III‐tubulin (ABclonal, A17913, diluted 1:1000); anti‐Influenza (Merck Millipore, AB1074, diluted 1:1000); anti‐Grx1, anti‐MMP‐9, anti‐M2, anti‐GAPDH (Santa Cruz Biotechnology, sc‐293250, sc‐393859, sc‐32238, sc‐47724, respectively, all diluted 1:1000); anti‐GSH (Virogen, 101‐A, diluted 1:500); anti‐Prdx1 (Abcam, 41906, diluted 1:1000); anti‐MMP‐2 (Bioss Antibodies, bs‐4599R, diluted 1:1000); anti‐Actin (Sigma‐Aldrich, A2066, diluted 1:1000); anti‐goat, anti‐mouse, anti‐rabbit (Bethyl Laboratories, A50‐101P, A120‐101P, A90‐116P, respectively, all diluted 1:10000). Detection was done with WesternBright ECL HRP substrate (Advansta) and densitometry by ImageJ/Fiji v2.14.0 (National Institutes of Health, United States).

### 2.7. Gelatin Zymography

To assess MMP‐2 and MMP‐9 activity, precipitated proteins from supernatants of differentiated and infected cells, collected at 8 and 24‐h post‐infection (p.i), were resolved on 10% SDS‐PAGE containing gelatin. Gels were washed, incubated with collagenase buffer and stained with Comassie Brilliant blue solution. Once destained, zymograms were visualized and acquired under visible light.

### 2.8. RNA Extraction and Quantitative Reverse Transcription‐PCR

Total RNA was extracted from cells with TRIzol (Sigma‐Aldrich) and quantified via NanoDrop (A260/280 = 1.9–2.1). mRNA was normalized to actin using the 2^−*ΔΔ*Ct^ method. Reverse‐transcription and quantitative PCR were performed using AMPLilabTM Real‐Time PCR System (Adaltis, Rome, Italy). Viral load was measured using SuperScript III qRT‐PCR kits (Life Technologies), quantified against plasmid standards and expressed in Log10 copies/mL [26].

All primer sequences for PCR are listed in Table S1.

### 2.9. ELISA

TNF*α* levels in culture supernatants, collected at 24‐h postinfection, were measured by ELISA according to the manufacturer′s instructions (Enzo).

### 2.10. MMP Silencing

Differentiated SH‐SY5Y cells were transfected with siRNAs targeting MMP‐2 and MMP‐9 or with a nontargeting control siRNA (siTOOLs Biotech) using Lullaby transfection reagent, according to the manufacturer′s instructions (Oz Biosciences), for 18 h. Silencing efficiency was assessed by western blot. Cells were subsequently infected with IAV as described above.

## 3. Statistical Analysis

Statistical analyses were performed using GraphPad Prism Version 9 (GraphPad Software). Data are presented as mean ± standard deviation (SD) from at least three independent experiments. For comparisons between two groups, a two‐tailed Student′s *t*‐test was applied. Comparisons involving multiple groups were analyzed using one‐way ANOVA followed by Dunnett′s post hoc test. Statistical significance was set at *p* < 0.05.

## 4. Results

### 4.1. NWS Virus Infects and Replicates in Differentiated SH‐SY5Y

**Figure 1:**
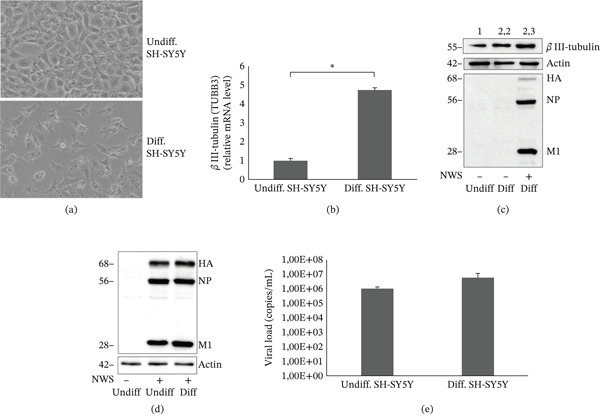
NWS virus infects and replicates in differentiated SH‐SY5Y cells. (a) Phase‐contrast microscope images of undifferentiated (undiff., above) and differentiated (diff., below) SH‐SY5Y cells after 6 days of retinoic acid (RA) treatment. Differentiated cells exhibit neurite outgrowth and a neuronal‐like phenotype. (b) Relative mRNA expression of the neuronal marker *β*III‐tubulin (TUBB3) in undifferentiated and RA‐differentiated SH‐SY5Y cells, assessed by qRT‐PCR. Data are presented as mean ± SD of three independent experiments; *p* < 0.01 (Student′s *t*‐test). (c) Western blot analysis of TUBB3 in undifferentiated (first lane) and RA‐differentiated, not infected (second lane) and NWS virus‐infected (third lane) SH‐SY5Y cells, 24‐h post‐infection (h p.i.). Actin was used as loading control. Densitometric analysis is reported above TUBB3 image, with values expressed as ratio TUBB3/actin. Blot was stripped and reprobed with anti‐influenza antibody which recognized viral proteins (HA, NP, and M1). Molecular weight of each protein is reported on the left of the blot. (d) Western blot analysis of viral proteins, in undifferentiated (second lane) and differentiated (third lane) SH‐SY5Y cells infected with NWS virus (MOI 0.1) at 24 h p.i, with anti‐influenza antibody. Actin served as loading control. (e) Viral load in supernatants from NWS‐infected undifferentiated and differentiated SH‐SY5Y cells 24 h p.i., measured by qRT‐PCR. Data are shown as mean ± SD (*n* = 3).

Human neuroblastoma cell line, SH‐SY5Y, was differentiated into a neuronal phenotype (Figure 1a), according to the protocol described in Methods. Differentiation was assessed morphologically and further confirmed at the molecular level by a significant increase in the neuronal marker *β*III‐tubulin (TUBB3) expression, as determined by qRT‐PCR (*p* < 0.01) and by western blot analysis (Figure 1b,c). Densitometry of *β*III‐tubulin normalized to actin revealed an increase in more than twice the protein expression in differentiated SH‐SY5Y cells, infected or not with NWS virus for 24 h, compared with undifferentiated cells. To verify the susceptibility of differentiated cells to NWS, western blot was stripped and reprobed with anti‐influenza antibody which recognized viral proteins hemagglutinin (HA), nucleoprotein (NP), and Matrix 1 (M1) (Figure 1c).

Western blot analysis of viral proteins in cell lysates was performed also on both undifferentiated SH‐SY5Y cells, which display an epithelial‐like phenotype, and differentiated SH‐SY5Y cells, characterized by a neuronal‐like phenotype. The results demonstrated the expression of the main viral proteins in both cell types, as shown in Figure 1d. Furthermore, the measurement of viral load in cell supernatants by qRT‐PCR confirmed that NWS efficiently infected and replicated in both undifferentiated and differentiated SH‐SY5Y cells. Specifically, viral load reached 1 × 10^6^ copies/mL in undifferentiated cells, whereas a slightly higher viral load of 6 × 10^6^ copies/mL was detected in differentiated cells (Figure 1e).

### 4.2. NWS Induces Significant Redox Changes

**Figure 2:**
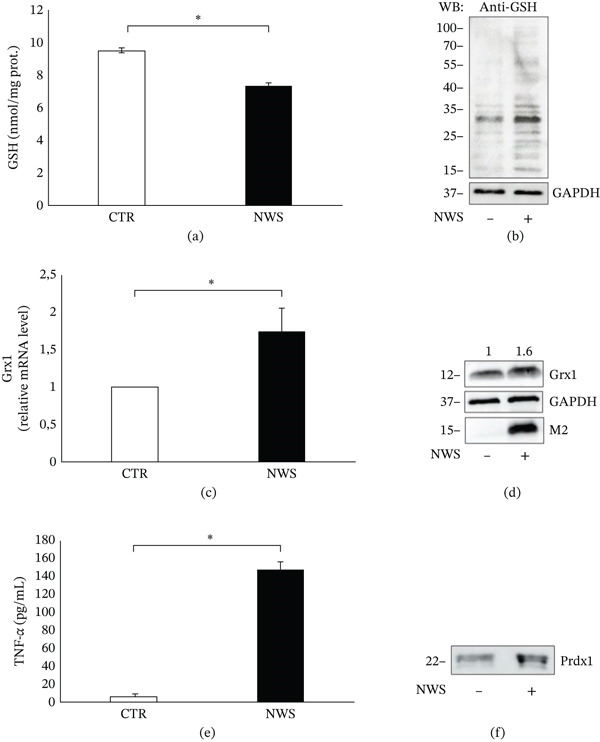
NWS infection induces significant redox changes in differentiated SH‐SY5Y cells. (a) Intracellular glutathione (GSH) levels in NWS‐infected differentiated SH‐SY5Y cells were measured 24 h p.i. GSH content was determined using a colorimetric assay and normalized to total protein content (nmol GSH/mg protein). Data are shown as mean ± SD (*n* = 3). Statistical significance was assessed using a two‐tailed Student′s *t*‐test; *p* < 0.05 versus control. (b) Glutathionylated proteins were detected in differentiated and infected SH‐SY5Y cells by western blot analysis with anti‐GSH antibody, following SDS‐PAGE in nonreducing conditions. GAPDH was used as loading control. (c) Grx1 mRNA levels, in differentiated and infected SH‐SY5Y cells, were quantified by qRT‐PCR, normalized to actin mRNA levels and expressed as fold change relative to control cells. Data represent mean ± SD (*n* = 3). *p* < 0.05 versus control. (d) Grx1 protein expression in the same samples of (C) was assessed by western blot. GAPDH was used as loading control. Densitometric values relative to GAPDH are reported above image. The blot was stripped and reprobed with viral anti‐M2 protein as infection control. (e) TNF*α* levels were measured in supernatants of differentiated and NWS‐infected SH‐SY5Y cells 24 h p.i. by ELISA. Data represent mean ± SD (*n* = 3). *p* < 0.05 versus control. (f) Western blot analysis of Prdx1 was performed in the same supernatants of (e).

Due to glutathione (GSH) fundamental role in maintaining redox homeostasis and protecting against oxidative stress, the intracellular GSH levels were measured in differentiated SH‐SY5Y infected cells. As showed in Figure 2a, a significant decrease in GSH levels was observed at 24‐h p.i. in comparison with noninfected cells. In the meanwhile, glutathionylated proteins, that is, mixed disulfides between GSH and protein cysteines, increased with infection (Figure 2b).

In addition to GSH and glutathionylated proteins, the expression of Glutaredoxin 1 (Grx1), a key thiol‐disulfide oxidoreductase involved in redox regulation by catalyzing the deglutathionylation of proteins, was quantified at 24 h p.i. The obtained results indicated a moderate but significant increase in Grx1 mRNA levels (Figure 2c), to which corresponded an enhanced protein expression (Figure 2d). To further analyze redox state, a redoxin, Peroxiredoxin 1 (Prdx1), previously shown to be released in oxidative stress conditions and acting as a danger signal [7, 5], was analyzed in supernatants of NWS‐infected cells along with a classical cytokine, TNF*α*. As shown in Figure 2e,f, infected cells released higher level of Prdx1, along with TNF*α*.

### 4.3. NWS Induces Redox‐Dependent Activation of MMPs

**Figure 3:**
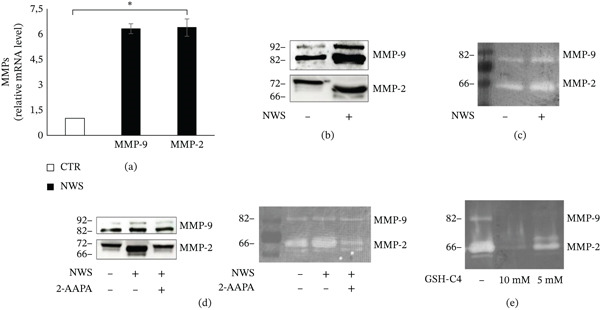
NWS virus induces MMP‐2 and MMP‐9 expression and secretion in differentiated SH‐SY5Y cells. (a) Relative mRNA expression levels of MMP‐2 and MMP‐9 were assessed in NWS‐infected differentiated SH‐SY5Y cells 24 h p.i. Expression was quantified by qRT‐PCR, normalized to actin, and expressed as fold change relative to uninfected controls. Data represent mean ± SD (*n* = 3). *p* < 0.05 versus control (Student′s *t*‐test). (b) Western blot analysis of supernatants from NWS‐infected differentiated SH‐SY5Y cells at 24 h p.i. showing the proMMPs (higher bands) and the active forms (lower bands) of MMP‐2 and MMP‐9. (c) Gelatin zymography of the same supernatants confirming the enzymatic activity of MMP‐2 and MMP‐9. (d) Western blot and gelatin zymography of NWS‐infected differentiated SH‐SY5Y cells treated with the redox modulator 2‐AAPA (100 *μ*M) 20 min before infection. Supernatants were collected and analyzed 24 h p.i. (e) Gelatin zymography of NWS‐infected differentiated SH‐SY5Y cells treated postinfection with the glutathione derivative GSH‐C4 (5 and 10 mM). Samples were collected 24 h p.i. (b–e) Representative images from three independent experiments are shown.

Oxidative stress plays a role in inducing the production of MMPs. In this context, using differentiated and infected SH‐SY5Y cells, the expression levels of MMP mRNAs were assessed and a significant upregulation of MMP‐2 and MMP‐9 was revealed (Figure 3a).

Subsequently, the expression of secreted endopeptidases was evaluated through western blot analysis of the supernatants and as shown in Figure 3b, the active form of the enzyme (lower band) was clearly observed. Through gelatin zymography, digestion bands in the supernatants of differentiated and infected SH‐SY5Y cells were detected. These bands correspond to the enzymatic activity of secreted endopeptidases (Figure 3c). At an earlier time point (8 h p.i.), zymography revealed detectable but modest induction of MMP‐2 and MMP‐9 activity compared with control cells (Figure S1), consistent with a progressive increase in MMP activation during infection.

To verify that the activation of these endopeptidases is redox‐regulated, SH‐SY5Y cells were treated with the redox modulator 2‐AAPA, a chemical inhibitor of Grx1. The results demonstrated that both the secretion and activation of MMPs significantly decreased (Figure 3d). Finally, to further confirm the redox‐dependence, a glutathione derivative with antioxidant properties, GSH‐C4, was used, observing a dose‐dependent inhibition of MMPs activation (Figure 3e).

### 4.4. Batimastat Inhibits Viral Replication

BB‐94 is a well‐known, potent broad‐spectrum inhibitor of MMPs that works by binding to the catalytic zinc ion in the active site of MMPs, thereby preventing their enzymatic activity [27].

Initially, the inhibitory capability of BB‐94 was verified in the SH‐SY5Y cellular model. As shown by zymography, the characteristic digestion bands (Figure 3c) indicative of MMPs activity were no longer visible upon BB‐94 treatment, confirming its effective inhibition of the catalytic site of these enzymes (Figure S2).

**Figure 4:**
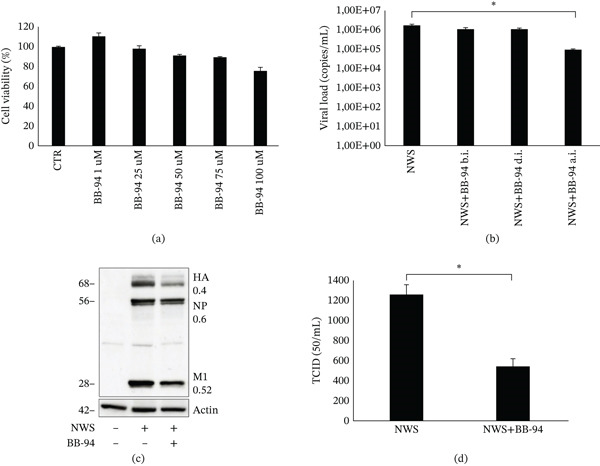
Effect of BB‐94 on viral replication and infectivity in differentiated SH‐SY5Y cells. (a) Cell viability of differentiated SH‐SY5Y cells treated with BB‐94 at concentrations ranging from 1 to 100 *μ*M for 24 h and assessed by MTT assay. Data are expressed as percentage relative to vehicle‐treated control cells. Data are shown as mean ± SD (*n* = 3). (b) Viral load quantified by qRT‐PCR in culture supernatants from cells treated with BB‐94 (25 *μ*M) at different stages of infection: before infection (b.i.), during infection (d.i.), or after infection (a.i.), compared with untreated infected cells. Supernatants were collected 24 h p.i. Data are shown as mean ± SD (*n* = 3). Statistical analysis was performed using one‐way ANOVA followed by Dunnett′s post hoc test versus NWS. (c) Western blot analysis of viral proteins (HA, NP, and M1) in cell lysates collected 24 h p.i. from infected cells treated postinfection with BB‐94 compared with untreated infected cells. Actin served as loading control. Numbers on the right represent densitometric ratios (viral protein/actin) expressed vs untreated values set as 1. (d) Infectious viral titers (TCID50/mL) measured from culture supernatants of BB‐94‐treated and untreated infected cells 24 h p.i. Data are mean ± SD (*n* = 3); *p* < 0.05 (Student′s *t*‐test).

In the meantime, to exclude cytotoxic effects of BB‐94, cell viability was assessed by MTT assay. Differentiated SH‐SY5Y cells were treated with increasing concentrations of BB‐94 (1–100 *μ*M) and, as shown in Figure 4a, cell viability was > 90% up to 50 *μ*M and of 98% at 25 *μ*M. Based on these results, all subsequent infection experiments were performed using BB‐94 at a concentration of 25 *μ*M. Therefore, differentiated SH‐SY5Y cells were treated with BB‐94 at different stages of infection: before infection with NWS strain, during infection, and after infection per 24‐h multicycle period.

Viral replication analysis indicated that BB‐94 treatment before and during infection resulted in similar viral titers, suggesting no significant effect at these stages (Figure 4b). In contrast, treatment after infection led to approximately a 1‐log significant reduction in viral replication (Figure 4b).

Moreover, analysis of viral protein levels also showed a reduction with BB‐94 treatment (Figure 4c), and this was accompanied by a decrease in infectious capacity, measured as TCID_50_ in the supernatants of treated samples (Figure 4d).

### 4.5. MMPs Silencing Reduces NWS Replication

**Figure 5:**
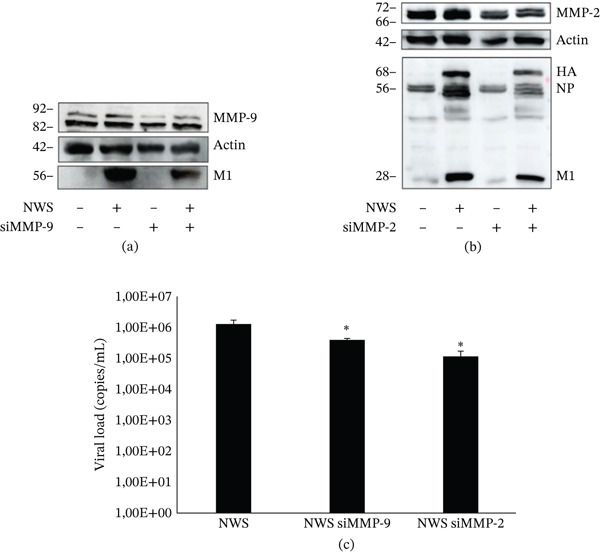
Effect of MMP‐2 and MMP‐9 silencing on NWS replication in differentiated SH‐SY5Y cells. (a) Western blot analysis of MMP‐9 protein levels in differentiated SH‐SY5Y cells transfected with control siRNA or siRNA targeting MMP‐9 and infected with the NWS strain. Actin served as a loading control. Blot was stripped and reprobed with anti‐influenza antibody. (b) Western blot analysis of MMP‐2 protein levels in differentiated SH‐SY5Y cells transfected with control siRNA or siRNA targeting MMP‐2 and infected with the NWS strain. Actin served as a loading control. Blot was stripped and reprobed with anti‐influenza antibody. (c) Viral RNA levels quantified by RT‐qPCR in culture supernatants from control and MMP‐2‐ and MMP‐9‐silenced cells infected with the NWS strain. Supernatants were collected 24 h p.i. Data are shown as mean ± SD (*n* = 3). Statistical analysis was performed using one‐way ANOVA followed by Dunnett′s post hoc test versus control siRNA

To evaluate the role of MMPs on NWS replication, MMP‐2 and MMP‐9 were silenced in differentiated SH‐SY5Y cells using specific siRNAs. The efficiency of gene silencing was assessed at the protein level by western blot analysis. As shown in Figure 5a and Figure 5b, protein levels of MMP‐9 and MMP‐2 were reduced in cells treated with the respective siRNAs compared with control cells of about 50%. The effect of MMP silencing on viral infection was then evaluated by assessing viral replication. Western blot analysis of viral protein expression showed decreased levels of viral proteins in MMP‐silenced cells compared with control cells (Figure 5a,b). Consistently, RT‐qPCR analysis showed a significant reduction in viral production in cells silenced for MMP‐2 and MMP‐9 compared with control cells (Figure 5c).

## 5. Discussion

IAV primarily targets epithelial cells of the respiratory tract, with viral subtypes exhibiting a differentiated tropism for respiratory regions, mainly determined by receptor expression: subtypes that infect the upper respiratory tract primarily interact with *α*2,6‐linked sialic acid receptors, whereas those infecting the lower respiratory tract prefer binding to *α*2,3‐linked sialic acid receptors [28]. Additionally, the temperature and pH conditions of various respiratory regions further influence viral stability and ability to infect target cells [29]. As receptor differences within the same cell type can influence the tropism of different subtypes of IAV, other cell types, such as those of the CNS, through several host and viral factors, might affect the efficiency of viral infection and replication [30]. The literature describes that neurotropic microorganisms can reach the CNS by overcoming the BBB through various mechanisms, including infection of circulating cells such as monocytes/macrophages [31]. Furthermore, for respiratory viruses, there may be an alternative pathway, often described as a “shortcut” to access the CNS. The influenza virus can directly infect the olfactory mucosa, with olfactory receptor neurons which can transport the virus via anterograde axonal transport, allowing the pathogen to reach the olfactory bulb [32]. Additionally, the virus can also infect supporting cells lining the channels of the ethmoid bone plate, with the potential for direct spread into the CNS [32, 33]. Viral invasion of the CNS can cause direct damage, through cytopathy of neuronal cells, or indirect damage, through immune‐mediated mechanisms that can lead to encephalitis. Although influenza encephalitis is a rare complication, it is a severe and potentially life‐threatening condition [4]. Beyond acute effects, recent studies suggest that chronic or subclinical CNS infection by influenza viruses may contribute to cumulative damage, facilitating the development of neurodegenerative disorders. This is supported by accumulating evidence linking chronic viral infections with neuroinflammation and persistent neuronal damage [34].

Many intracellular pathways are involved in the response to influenza infection in target cells, including redox‐regulated signaling pathways that can induce the production of MMPs [35]. This process can significantly impact both viral propagation and the pathogenesis of inflammatory complications [36, 37].

Although these mechanisms have been extensively described in epithelial cells, much less is known about their activation and function in neuronal cells.

This knowledge gap forms the basis of our study, which is aimed at developing an in vitro model using differentiated human neuroblastoma SH‐SY5Y cells to investigate IAV infection within the CNS. We specifically aimed to better understand the mechanisms underlying infection in neuronal‐like cells, focusing on the induction of oxidative stress by the infection and the impact of the virus on the production of MMPs.

For this reason, as a first objective, a reliable and reproducible in vitro model was developed using differentiated human neuroblastoma SH‐SY5Y cells to investigate IAV infection within the CNS. Differentiated SH‐SY5Y cells are widely recognized as a valuable in vitro model to investigate neuronal processes and neuropathogenic mechanisms [22].

Our results confirm that Influenza A/NWS/33 H1N1 (NWS) strain can infect and replicate in differentiated SH‐SY5Y neuronal‐like cells as demonstrated by the expression of viral proteins and viral load analysis.

A crucial aspect of our study was the analysis of redox state alterations in response to viral infection. We observed a significant decrease in GSH levels in infected SH‐SY5Y cells and an increase in glutathionylated proteins. This finding is consistent with the fact that many viral infections, including influenza, induce oxidative stress in host cells, with IAV replication efficiency being dependent on GSH levels [23]. Although these findings were primarily described in nonneuronal cells, our study expands this knowledge by demonstrating that IAV also exploits oxidative stress to sustain replication in differentiated SH‐SY5Y neuronal‐like cells. In addition, the increased expression of Grx1 in infected cells suggests a compensatory attempt to maintain redox homeostasis under oxidative stress conditions induced by the infection [38]. Moreover, the analysis of cell supernatants revealed the release of a redoxin, Prdx1, previously shown to act as a danger signal [39], along with a canonical cytokine, TNF*α*.

Another essential process for viral propagation is the production of MMPs. Our results show that infection induces a significant increase in MMP‐2 and MMP‐9 expression, accompanied by the activation of their proteolytic activity. Our analysis indicated that the activation of MMPs is redox‐regulated. Specifically, the use of redox modulators such as the Grx1 inhibitor 2‐AAPA and a glutathione derivative GSH‐C4 showed a significant reduction in MMPs activation. This suggests that oxidative stress plays a crucial role in the activation of these endopeptidases. The dose‐dependent inhibition observed with GSH‐C4 could suggest that the intracellular redox state directly controls MMPs activation.

The roles of host proteases in the enveloped virus replication cycle are extensively documented [40].

The paper by Matarrese et al. demonstrated that the inhibition of specific lysosomal proteases, such as Cathepsin D by Pepstatin A, can hinder the replication of IAV by modulating the cell autophagic machinery [41]. The finding fits into a broader context of studies showing how viruses can manipulate host proteases and related pathways to optimize their replication.

Recent research on SARS‐CoV‐2 has demonstrated that this virus, particularly its variants of concern such as Delta, can exploit MMPs to enter the cells and for cell‐to‐cell fusion; in fact, MMP‐2 and MMP‐9 can activate the viral glycoprotein S in a TMPRSS2 and cathepsin‐independent manner via entry in cells expressing high MMPs level [42].

Given our results on a significant increase in MMP‐2 and MMP‐9 expression and activity in NWS‐infected neuronal‐like cells, it is plausible to speculate that a similar protease‐dependent mechanism could play a role in influenza glycoprotein activation.

In the present study, the effect of MMP on viral replication was explored through the approaches of chemical inhibition, using BB‐94, and of downmodulation by siRNA. Our results show that BB‐94 treatment after infection, as well as MMPs downmodulation, significantly reduces viral replication. The finding that BB‐94 significantly reduces viral replication and the infectivity of viral particles only when administered after infection suggests that MMPs′ activity may be involved in postentry stages of the viral life cycle, such as viral maturation, release, or cell‐to‐cell spread, rather than in viral entry or cell susceptibility. Although the precise mechanisms remain to be elucidated, these results highlight a potential role for MMPs in supporting later phases of IAV replication.

The role of MMPs in both SARS‐CoV‐2 and IAV infections may highlight a conserved strategy among respiratory viruses, whereby modulation of host proteolytic pathways, particularly through MMPs, may enhance viral propagation and exacerbate tissue damage. Although our study did not directly investigate these mechanisms, the significant reduction in viral replication and infectivity observed upon broad MMP inhibition supports a functional role of MMPs in promoting IAV propagation in neuronal‐like cells. Since this model does not fully recapitulate the properties of mature neurons, future studies in more complex systems, such as primary neurons or brain organoids, will be critical to establish the biological relevance of these observations.

In conclusion, our findings provide compelling evidence that IAV can infect neuronal‐like cells, specifically differentiated SH‐SY5Y cells and induce alterations in the redox state and activation of MMPs, highlighting important cellular processes that may be exploited by the virus to facilitate replication and spread and offer promising therapeutic strategies for limiting viral replication and associated tissue damage. These insights underline the complex interplay between viral infection and cellular responses in neuronal environments.

## Author Contributions

**Carla Prezioso**: conceptualization, data curation, investigation, methodology, validation, visualization, writing—original draft, writing—review and editing. **Marta De Angelis:** investigation, writing—review and editing. **Dolores Limongi**: resources. **Anna Maria Marinelli**: investigation. **Flavio Frezza:** investigation. **Lucia Nencioni**: supervision, writing—review and editing. **Anna Teresa Palamara**: supervision, writing—review and editing. **Paola Checconi**: conceptualization, data curation, investigation, methodology, validation, visualization, writing—original draft, writing—review and editing, funding acquisition. Anna Teresa Palamara and Paola Checconi contributed equally to the work.

## Funding

This study was supported by Ministero dell′Istruzione, dell′Università e della Ricerca (10.13039/501100003407, MUR PRIN 2017KM79NN , MUR PRIN 2022HARH5W); Ministero della Salute (10.13039/501100003196, Ricerca corrente).

## Conflicts of Interest

The authors declare no conflicts of interest.

## Supporting information


**Supporting Information** Additional supporting information can be found online in the Supporting Information section. Table S1 Primer sequences for qRT‐PCR. Figure S1: Early MMPs activity at 8 h postinfection. Gelatin zymography showing MMP‐2 and MMP‐9 activity in supernatants from differentiated and NWS‐infected SH‐SY5Y cells 8 h post‐infection (p.i.). Infected samples show detectable but modest induction of MMP‐2 and MMP‐9 activity compared with uninfected controls (respectively, 1.3 and 1.2 fold vs. uninfected controls). Figure S2: Inhibition of MMPs catalytic site by BB‐94. Gelatin zymography of culture supernatants from differentiated and NWS‐infected SH‐SY5Y cells 24 h p.i., resolved on 10% SDS‐PAGE containing gelatin, and the part on the right incubated with BB‐94 (50 *μ*M). The digestion bands, indicative of MMPs activity (left of the zymography) were no longer visible upon BB‐94 treatment (right of the zymography), confirming its effective inhibition of the catalytic site of these enzymes.

## Data Availability

The data that support the findings of this study are available from the corresponding author upon reasonable request.
