# Supplementary material for: Redox‐Dependent Activation of Matrix Metalloproteinases Regulates Influenza A Virus Replication in Neuronal Cells
Source: Int J Microbiol. 2026 May 18;2026:7586794. doi: 10.1155/ijm/7586794 (PMC13181273; doi:10.1155/ijm/7586794)
Supplement: Supplementary file 1 — Supporting Information Additional supporting information can be found online in the Supporting Information section. Table S1 Primer sequences for qRT‐PCR. Figure S1: Early MMPs activity at 8 h postinfection. Gelatin zymography showing MMP‐2 and MMP‐9 activity in supernatants from differentiated and NWS‐infected SH‐SY5Y cells 8 h post‐infection (p.i.). Infected samples show detectable but modest induction of MMP‐2 and MMP‐9 activity compared with uninfected controls (respectively, 1.3 and 1.2 fold vs. uninfected controls). Figure S2: Inhibition of MMPs catalytic site by BB‐94. Gelatin zymography of culture supernatants from differentiated and NWS‐infected SH‐SY5Y cells 24 h p.i., resolved on 10% SDS‐PAGE containing gelatin, and the part on the right incubated with BB‐94 (50 μM). The digestion bands, indicative of MMPs activity (left of the zymography) were no longer visible upon BB‐94 treatment (right of the zymography), confirming its effective inhibition of the catalytic site of these enzymes. [file IJM-2026-7586794-s001.docx]

| **Gene** | **Direction** | **Sequence (5′→3′)** |
| --- | --- | --- |
| **HA** | Forward | TGGGGCCATTGCCGGTTTCA |
|  | Reverse | TGCCCCCAGGGAGACTACCA |
| **MMP2** | Forward | CTTCCAAGTCTGGAGCGATGT |
|  | Reverse | TACCGTCAAAGGGGTATCCAT |
| **MMP9** | Forward | GGGACGCAGACATCGTCATC |
|  | Reverse | TCGTCATCGTCGAAATGGGC |
| **GRX1** | Forward | GGAGCAAGAACGGTGCCTCGAG |
|  | Reverse | AAAGCAGATTGGAGCTCTGCAG |
| **ACTB (β-actin)** | Forward | AGCGTCTTGTCATTGGCGAA |
|  | Reverse | TTTTCTGCTCCAGGCGGACT |
| **TUBB3** | Forward | CAACCAGATCGGGGCCAAGTT |
|  | Reverse | GAGGCACGTACTTGTGAGAAGA |

**Table 1.** Primer sequences for qRT-PCR.


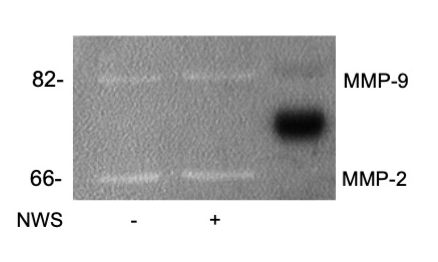


**Figure S1. Early MMPs activity at 8 h post-infection.** Gelatin zymography showing MMP-2 and MMP-9 activity in supernatants from differentiated and NWS-infected SH-SY5Y cells 8 h post-infection (p.i.). Infected samples show detectable but modest induction of MMP-2 and MMP-9 activity compared to uninfected controls (respectively, 1.3 and 1.2 fold *vs* uninfected controls).


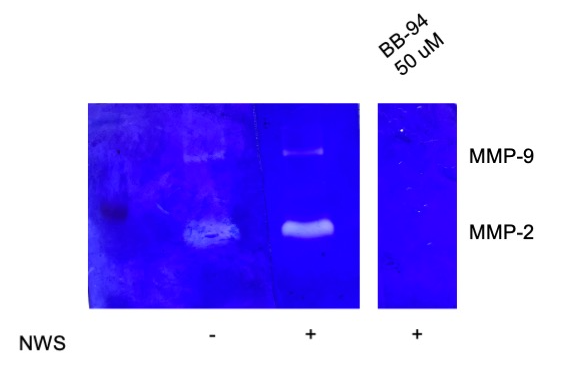


**Figure S2. Inhibition of MMPs catalytic site by BB-94**. Gelatin zymography of culture supernatants from differentiated and NWS-infected SH-SY5Y cells 24 h p.i., resolved on 10% SDS-PAGE containing gelatin, and the part on the right incubated with BB-94 (50 μM). The digestion bands, indicative of MMPs activity (left of the zymography) were no longer visible upon BB-94 treatment (right of the zymography), confirming its effective inhibition of the catalytic site of these enzymes.
